# A Method for Evaluating and Selecting Suitable Hardware for Deployment of Embedded System on UAVs

**DOI:** 10.3390/s20164420

**Published:** 2020-08-07

**Authors:** Nicolas Mandel, Michael Milford, Felipe Gonzalez

**Affiliations:** 1 Australian Centre of Excellence for Robotic Vision, Queensland University of Technology, Brisbane QLD 4000, Australia; michael.milford@qut.edu.au (M.M.); felipe.gonzalez@qut.edu.au (F.G.); 2 QUT Centre for Robotics, Queensland University of Technology, Brisbane QLD 4000, Australia

**Keywords:** UAV, computer architecture, decision making, navigation, semantics

## Abstract

The use of UAVs for remote sensing is increasing. In this paper, we demonstrate a method for evaluating and selecting suitable hardware to be used for deployment of algorithms for UAV-based remote sensing under considerations of *Size*, *Weight*, *Power*, and *Computational* constraints. These constraints hinder the deployment of rapidly evolving computer vision and robotics algorithms on UAVs, because they require intricate knowledge about the system and architecture to allow for effective implementation. We propose integrating computational monitoring techniques—profiling—with an industry standard specifying software quality—ISO 25000—and fusing both in a decision-making model—the analytic hierarchy process—to provide an informed decision basis for deploying embedded systems in the context of UAV-based remote sensing. One software package is combined in three software–hardware alternatives, which are profiled in hardware-in-the-loop simulations. Three objectives are used as inputs for the decision-making process. A Monte Carlo simulation provides insights into which decision-making parameters lead to which preferred alternative. Results indicate that local weights significantly influence the preference of an alternative. The approach enables relating complex parameters, leading to informed decisions about which hardware is deemed suitable for deployment in which case.

## 1. Introduction

Unmanned Aerial Vehicles (UAVs) are playing an increasingly important role in modern society. The past decade has seen an increased use of UAVs, with applications ranging from wildlife and agricultural monitoring [1,2,3], over racing [4], industrial monitoring [5], packet delivery [6], and search and rescue [7] to planetary exploration [8]. Solutions to computer vision problems, such as object detection [9], and robotics problems, such as localization [10], have been developed and disseminated at a rapid pace, with promising results on benchmarks [11]. Despite experiments on real robots being the common mode of evaluation for robotics applications [11], reproducing results requires a considerable effort [12]. Furthermore, computational performance has traditionally been considered a secondary factor [10], assuming that hardware progress will eliminate this issue.

At the same time, Wirth’s law states that the increasing complexity in software outgrows the increase in computational power. Additionally, UAVs are heavily constrained through their *Size*, *Weight*, *Power*, and *Computation* [6,13,14,15]. The main limitation for small multirotor UAVs is their flight time, which is bound by the battery capacity, which is in turn limited by the *Size* and *Weight* of the UAVs. Over 90% of *Power* is consumed by the motors supplying the thrust; however, the influence of computational capabilities on *Power* is complex. While increasing computational load uses more *Power* directly, faster computation can greatly reduce total power consumption for the same task due to reduced time spent at hover and lower accelerations, to up to 6 times faster completion of tasks [6].

**Figure 1:**
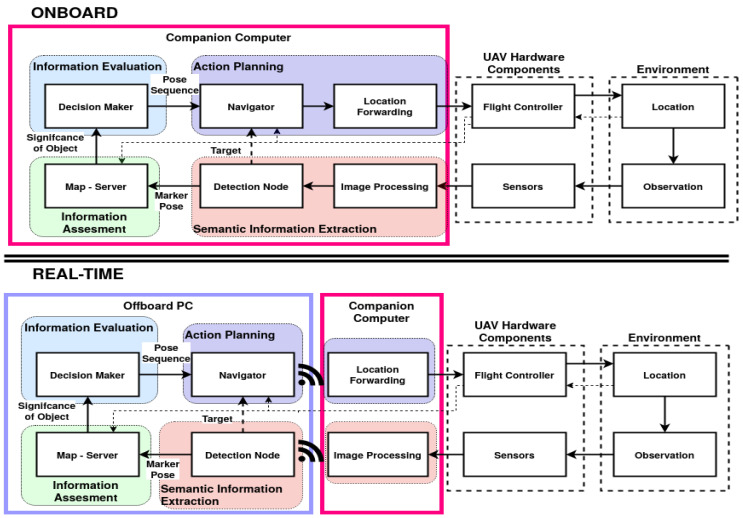
Modules developed in [35], onboard (**top**) and a real-time (**bottom**) *configuration*, with the system load distributed across a PC (purple) and an onboard computer (magenta). The thin dashed line indicates a serial connection and the wireless symbol a network connection. Differences in software set-up are called *configurations* in this work.

**Figure 2:**
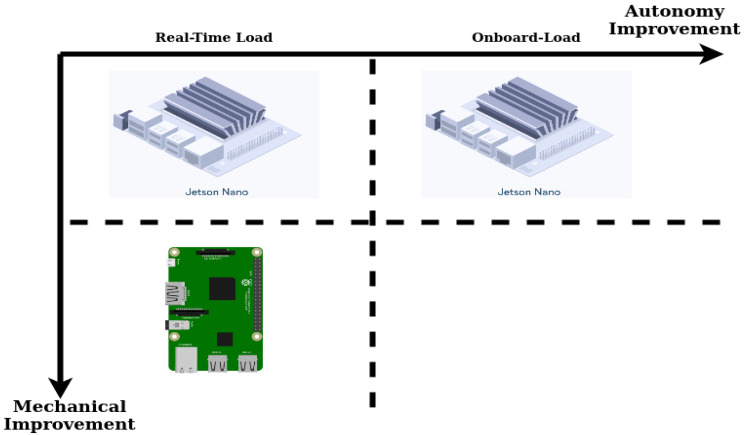
The three *alternatives* evaluated in this work. Differences in software *configurations* as shown in Figure 1 are shown on the *x*-axis. The *y*-axis denotes an additional mechanical variation through changing the companion computer.

Using the maximum computation available by offloading parts of the perception—planning—control loop over a network connection is the most common solution [6,16,17,18,19,20]. This method is highly effective, however it introduces networking effects and drop-outs, with potentially detrimental consequences when used in real-time cycles [21,22]. Furthermore, several remote-sensing applications cannot rely on network connections, making onboard computation the final goal. Some research has focused on the onboard application of algorithms [4,23,24,25], however most are the result of empirical processes. Only a finite amount of research is concerned with detailed evaluation of software and hardware performance [23,26,27]. Using a powerful laptop for computation is a possible solution, however *Size* and *Weight* do not always allow for such a payload. In this paper, the difference between onboard and real-time as illustrated in Figure 1 is termed *configuration*, while the combination of a software *configuration* and a specific hardware as illustrated in Figure 2 is termed *alternative*.

The question as to which hardware is considered as a suitable solution is therefore highly complex and requires consideration of mechanical, electrical, and computational constraints imposed on small UAVs. Boroujerdian et al. [6] uncovered the influence of computation on power consumption, while Krishnan et al. [27] connected mechanical and computational performance in a “roofline” model. However, to the best of our knowledge, no method exists that holistically combines and analyses all four of these factors together. To this end, a decision-making approach from mathematical economics, the analytic hierarchy process (*AHP*) [28], is proposed.

Additionally, the question as to which indicators underline the quality of software is posed. While fast computation reduces flight time in some cases, in others the benefit is marginal, especially when tasks do not require compute-intensive dynamic recalculation steps [6]. On the other hand, some UAVs may have supplementary tasks to fulfill, such as manipulation [29,30], with different needs, such as the capacity to accommodate other software. These diverging specifications call for a framework with a balance between flexibility and rigidity in definitions of quality and associated metrics. The use of the ISO 25000 family “Systems and Software Quality Requirements and Evaluation” (*SQUARE*) [31,32,33] is proposed to meet these requirements. Software development is an intricate process, especially for robotic systems, which are commonly evaluated in experiments and therefore require implementation on realistic hardware [11]. Standardized frameworks such as the Robot Operating System (*ROS*) are universal and alleviate issues with interoperability, however, reproducing results remains an issue [12]. Pinpointing bottlenecks when implementing a software on a hardware system is a difficult task and requires detailed profiling [34].

**Figure 3:**
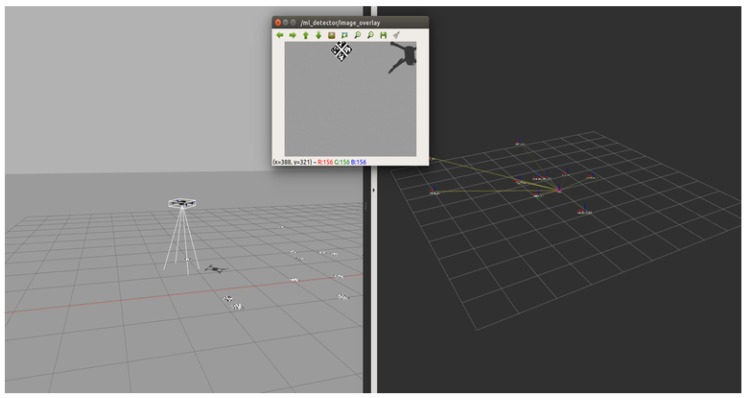
A software-in-the-loop (SITL) simulation in the Gazebo Environment is shown on the left. The UAV is conducting an autonomous remote-sensing search mission for a target marker. The top panel shows the view of the UAV; the right panel shows the map of detected markers.

In this work, we demonstrate the methods using a software-in-the-loop (SITL) ROS package developed and validated by the authors [35]. Figure 3 shows the simulation in the process. The left image shows the Gazebo simulation environment, the top panel shows the UAV view with a marker visible, and the right panel shows the reconstructed map.

The main driver of the subsequent hardware-in-the-loop (HITL) development is the preference of evaluation over experimentation as a factor of successful developments as put forward by Corke et al. [11]. Two embedded devices—a nVidia Jetson Nano and a Raspberry Pi 3A+—with deviating properties are evaluated. The Nano is computationally powerful, while incorporating a large volume and being heavier. The Pi is smaller, with less powerful computation.

Profiling techniques are used to inspect the computational performance of the software–hardware combination and are put into relation with one another using the *SQUARE* family. The computational performance is combined with electrical and mechanical factors through the AHP to provide numerical values which *alternative* is considered best.

Three case studies with different input weights for the AHP are proposed to verify the approach. The recorded parameters from the HITL simulation are used to show how the results change with varying the weights. Additionally, a Monte Carlo (MC) simulation is used over the admissible input space, which allows to compare the effects of different relative weights to give preference to another *alternative*.

Figure 1 presents one example of the differences between an onboard and a real-time system in the context of UAV onboard decision-making [35]. The split of the nodes is dependent on the frequency and the degree of closeness to hardware required by hardware components.

In this work, a methodical approach to HITL simulations is employed. The transition steps from SITL to HITL and their effects down the line of development are shown. A framework for evaluating the quality of software is proposed. Additionally, the AHP is drawn on for combining requirements along different dimensions, mainly mechanical and computational. Furthermore, to the best of our knowledge, the amount of research concerned with a systematic transition from simulation to experimental deployment is limited and we aim to augment the literature by providing an evaluation method.

## 2. Background

Evaluating system performance can be a slow, methodical task [34]. However, it is necessary to prevent detrimental effects down the line of development and deployment of UAVs. These can have catastrophic consequences when considering the deployment of robotic systems in critical conditions, such as near urban areas or oil rig inspection, where hardware failure may result in injuries to personnel on the ground or damage worth millions of dollars and years of work.

Models to evaluate the performance and potential of software on specific hardware are readily available. One example is the commonly known “roofline” model [36]. However, improvements implied by these models are gained through hardware-near programming techniques requiring profound knowledge of memory and CPU access. This is difficult to achieve for programmers working in a meta-environment such as ROS, which employs a complex variety of different tools in a software stack [12,37]. Models for evaluating the performance of GPUs in the context of contemporary deep learning approaches inspected the timeliness and performance [38] and the potential to be used with common embedded platforms [26]. The applicability of one of these algorithms on embedded platforms in the context of UAVs was evaluated by Kyrkou et al. [23] and subsequently a dedicated architecture solution was proposed [39].

The constraints on the computational capabilities of small multirotor UAVs caused by reduced availability of *Size*, *Weight*, and *Power* require a careful balance between computer hardware and algorithm efficiency [6,13,14,15]. Krishnan et al. [27] tied the computational performance together with mechanical properties of a UAV in the derivation of a modified “roofline” model [36] to provide hard limits on flying velocity. However, their model only considers obstacle avoidance as a task and neglects higher level navigation tasks [40]. While *Size*, *Weight*, and *Power* are relatively straightforward to approximate, *Computation* is more difficult to put into numbers. Tools to passively record the computational performance—commonly called “profiling” tools—are available in the scientific literature [22,23,26,27,36], as well as in industry [31,34,41]. However, the information gathered from these systems is either used to identify computational bottlenecks [34] or not used to make decisions at all. The use of the ISO 25000 SQUARE software model is proposed to relate computational performance indicators extracted from profiling.

### 2.1. The ISO SQUARE Family

The SQUARE family was published in the early 2010s [31]. It provides eight characteristics, each with varying sub-characteristics, on which to evaluate the quality of a software, and explicitly extends to embedded hardware–software systems. Which characteristics to use and at what point to define what is to be evaluated (e.g., at function calls, at server accesses, etc.) is up to the discretion of the developer, however it requires explicit definition. ISO 25021 defines *quality measure elements*, while ISO 25023 proposes some metrics on which to evaluate the sub-characteristics, as well as how to develop and combine these [33]. The analysis in this work is reduced to the following characteristics.

1. Performance Efficiency
2. Compatibility
3. Reliability

The remaining criteria of the ISO are *Functional Suitability*, *Usability*, *Security*, *Maintainability*, and *Portability*. ROS as a standardized framework that handles *Maintainability* and *Portability* [12]. *Functional Suitability*, *Usability*, and *Security* are equal across all *configurations* for the evaluated software and are therefore not considered in this work. If *alternatives* would display differences across these characteristics, they would require integration into the hierarchy and thus the analysis.

CPU load and memory are recorded to evaluate *Performance Efficiency* and *Compatibility*, as these are commonly considered the main determining performance factors [36]. *Resource Utilization* is the sub-characteristic of *Performance Efficiency* targeted in this research. *Time behavior* is not evaluated due to its inherent issues discussed in the literature [21,22]. GPU usage has been broadly evaluated in the literature [23,26,27], and as the evaluated application does not target it [35], it is omitted. *Capacity* is deemed not adequate to evaluate, because the system load only changes minutely over time and inputs cannot be scaled infinitely.

For *Compatibility*, the sub-characteristic *Interoperability* is equated through ROS. This research focuses on *Co-Existence*, which refers to the property of not negatively influencing other software running on the same system.

*Fault Tolerance* and *Recoverability* are sub-characteristics of *Reliability*, which are governed by the flight controller. *Availability* is a network property not considered adequate for the evaluation of the system; however, *Maturity* is evaluated through the number of faults.

Commonly, profiling is used as an approach to identify bottlenecks in the system [6,34]. However, bottlenecks reduce issues to a single part of the system and neither consider the impact on the entire system nor other dimensions beyond computation. The use of the AHP is proposed to alleviate this issue, which was developed by Thomas Saaty in the late twentieth century [28] and has been applied to a diverse set of decisions systems [42], including, but not limited to, medical [43], mining [44], flood susceptibility [45], and transfer learning [46] analyses.

## 3. Materials and Methods

### 3.1. Analytic Hierarchy Process

In the AHP, a hierarchy of criteria, termed levels in the following sections, is developed. The criteria at each level are compared against one another and assigned a weight between 1 and 9 to describe their relative importance [42]. The weights are commonly assigned subjectively by stakeholders, for example, through consensus voting [28] or sampling from expert opinions [45]. While other methods are available, the choice of weights is beyond the scope of this paper and discussed in [28,42,47]. To illustrate, *Power* is considered twice as important as *Weight* and *Weight* three times more important than *Size*. A matrix is constructed with these weights and its normalized maximal Eigenvector is calculated, which yields the local priority of each criterion. Only matrices with consistencies as calculated by Equation (1),

(1) $$CI=\frac{\lambda_{max}-n}{n-1}$$

with $\lambda_{max}$ as the maximum Eigenvalue and *n* the dimension of the matrix, are allowed, as some constellations are considered unrealistic. For example, if *Weight* is twice as important as *Size* and *Power* is three times as important as *Weight*, *Size* cannot be more important than *Power*, as this would violate transitivity. However, small inconsistencies, which are bound to arise due to the constrained scale, are allowed and therefore the Consistency Index CI is divided by an empirically calculated *R*, $CR=\frac{CI}{R}$, to result in a value which should be below 0.1 [28,43]. This procedure is repeated for each hierarchical level, where the global priority is calculated through multiplication with the priority of the subsuming characteristic from the next higher level, thus resulting in a hierarchy of priorities.

The *alternatives* are compared pairwise with respect to each of the criteria and result in a relative preference for either one of the *alternatives*. For example, if one *alternative* is twice as heavy as another one, its preference for weight will be 12. These preferences are then multiplied with the priorities to result in a priority for each criterion for each *alternative*.

**Figure 4:**
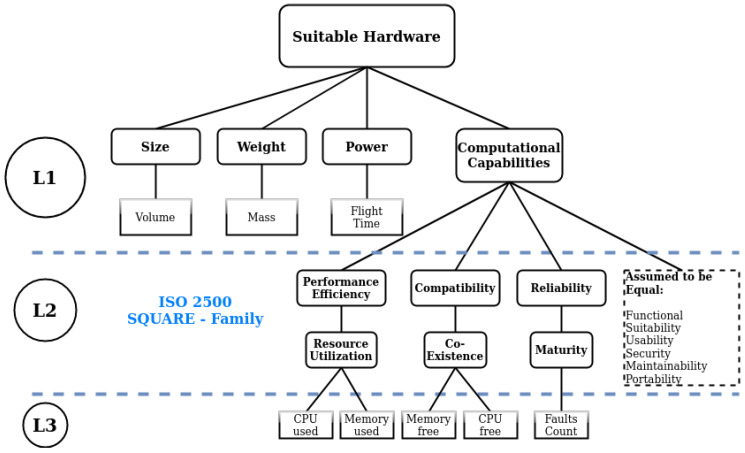
The final analytic hierarchy process (AHP) hierarchy used in the evaluation steps. The first level (L1) depicts the parameters common to literature [6]. The second level (L2) includes the parameters that are derived from the *SQUARE* family [31] and the third (L3) includes common computational parameters [33,36].

This method allows the hierarchical characteristics and sub-characteristics of the SQUARE family to be related to one another, as well as put into context with the criteria *Power*, *Weight*, and *Size* commonly mentioned in the literature [6,13,14]. It furthermore allows to evaluate discrete *alternatives* of embedded systems and put values into context with one another. Figure 4 shows the hierarchy developed and used in the following sections.

#### 3.1.1. Case Studies

The global preference of the available *alternatives* largely depends on the weights assigned to each of the criteria at the different levels of the hierarchy. Three cases inspired from remote sensing scenarios are used to derive weights for the levels of the AHP. These weights are used to calculate different priorities, which are then multiplied with the same preference values for the *alternatives* resulting from profiling as described in Section 4.2. Without loss of generality, the preference values from the same experiments are used to illustrate the influence of differing weights on final priority. The following three tasks are chosen.

1. A fully autonomous UAV performing oil rig inspection [5]
2. A UAV in a controlled environment with a suspended arm [29,30]
3. An additional module on a UAV to improve performance by integrating semantics into its navigation [35]

These cases lead to assumptions concerning the assignment of weights, which require revision until the matrices can be considered consistent as defined by Equation (1). The weight matrices are explained in detail in Section 4.5.

#### 3.1.2. Monte-Carlo Simulation

Each of the *alternatives* (presented in Figure 2) can result as the best *alternative* given certain input weights. A MC simulation is conducted to find these weights. The space of consistent weight matrices is searched for the most dominant results, and the relative weights are presented. The results collected from the initial experiments—detailed in Section 4.5 and shown in Table 1—are used as inputs for the MC simulation. Ten-thousand iterations are run to generate values for the upper right triangular matrix from the original range from 1 to 9 and their inverses f:S↦X,f(s)=1s, with D=X∪S, $\underline{D}=17$ admissible values [28]. Values from D are chosen at random to construct the upper right triangular matrix *M*, with the lower triangular matrix filled with the inverse, so $m_{j,i}=\frac{1}{m_{i,j}}\forall j>i$. The resulting matrix *M* is checked for consistency using Equation (1) and added to the set of admissible matrices only if it is consistent.

To clarify, for rnk(M)=2 this leads to 17 possible combinations, because there is only one degree of freedom, $m_{1,2}$, which can take all 17 values from the admissible set *D*. For 3 and 4, 1228 and 479 consistent matrices are saved, respectively. The set of applicable matrices is denoted with $Y_{i},i\in 2,3,4$. Algorithm 1 details the process on calculating the final score for each *alternative*. $M_{i}$ denotes a matrix from the set of consistent matrices. ${\hat{v}}_{j}$ the global priority after multiplication with the higher level j−1. *P* denotes the matrix of recorded values derived from Table 1 and Section 4.4 and $\hat{p}$ the final priority of the *alternative*. The 10,000 resulting global priorities $\hat{p}$ are stored along with their respective weight matrices, M. In a final step, the constellation from the global priorities which would have led to the clearest preference of a given *alternative* are calculated and the weight matrices for that option are extracted for qualitative inspection.
**Algorithm 1** Monte Carlo Simulation**for** 1 **to** 10,000 **do**
$M\leftarrow M_{j}\in Y_{i}$$v_{j}\leftarrow$ max. Eigenvector of $M_{j}$${\hat{v}}_{j}\leftarrow v_{j}\cdot {\hat{v}}_{j-1}$$\hat{p}\leftarrow P\cdot {\hat{v}}_{j}$**return**$\hat{p}$, *M*
**end for**

## 4. Experimental Design

**Figure 5:**
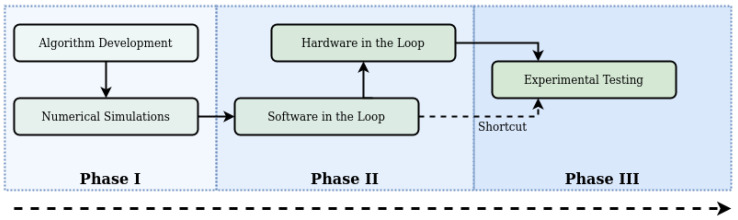
A generic autonomy development cycle, adapted from Ladosz et al. [48]. The arrow depicts the line of development, with the shading indicating the completeness. This work focuses on the transition included in Phase II.

This work is targeting the HITL development step shown in Figure 5. Algorithm development, numerical simulations, and SITL—shown in Figure 3—were conducted in previous research and the performance of the developed package was verified [35]. The current work focuses on translating the simulation with the package into a distributed set-up, with the simulation running on a host PC—connected via Ethernet to the router—and parts of the package—in variations shown in Figure 1—running on the embedded system. The flight controller is connected to each device with an independent serial connection. This way it communicates with the simulator as well as the embedded computer.

### 4.1. System Specifications

All development procedures are undertaken on a Dell Notebook with an nVidia GeForce GTX 1060 Max-Q graphics card and an Intel i9-8950HK Processor with 16 GB of RAM and 24 GB of Swap. Ubuntu 18.04 with ROS melodic is used alongside Gazebo-9 for the simulation and development. A mix of C++ and Python nodes are used during development. An open source node developed in-house is used for the image detection, while all other nodes were verified previously [35]. A vanilla version 10.1 of the Px4 Firmware on a PixHawk 4 is used. OpenCV version 3.4.6 with contrib modules is used for image processing. The network setup is run over a dedicated router within 1 m in line-of-sight, operating in the 5 to 2.4 GHz range to minimize network effects. The implementation details to enable reproduction [12] are detailed in Appendix A.

### 4.2. Development Steps

The simulation environment is shown in Figure 3. An UAV is exploring its environment and uses the camera feed to look for markers. If a certain type of marker is detected, the position is recorded and the path recalculated under consideration of known and unknown areas, as well as location of markers. Ten different marker placements, which were used for SITL [35], are used to record values according to Section 4.4 for each *alternative* shown in Figure 2.

**Figure 6:**
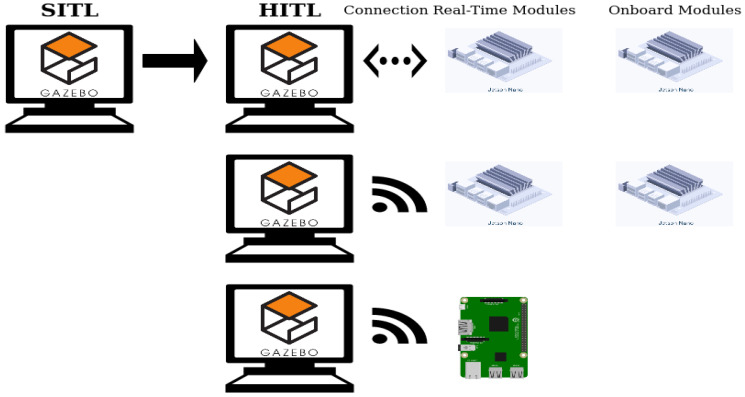
Hardware-in-the-loop set-ups. Each row shows changes in the hardware set-up and each column on the right changes in the software. The first row shows the transition from SITL to HITL using an Ethernet connection. The first column shows a real-time *configuration* as shown in Figure 1 and the the second column shows an onboard *configuration*. The second row shows the transition to a wireless connection. The third row shows the transition to the second embedded device.

Translating from SITL to HITL requires consideration of non-local communication. Packages are downloaded to the more powerful embedded device, the Jetson Nano. In a first step, the device is connected via Ethernet to the router, as shown in the first row of Figure 6, and one simulation is run with the real-time *configuration* of the package, as shown in Figure 1. Upon successful completion without notable errors or warnings, one simulation in onboard *configuration* is run, shown in the last column of Figure 6. When this test passes without notable errors, the network connection is switched to wireless and the test is repeated with both *configurations*, shown in the second row of Figure 6. When all tests are passed, the package is translated to the less powerful embedded device, the Raspberry Pi, as shown in the last column of Figure 6.

Each of these steps has an impact on the performance of the system, which may manifest itself in different ways and can be counteracted through adaptive software or hardware measures before continuing with the next step. The impact of these measures down the line of development should not be neglected, and therefore these steps are kept separate. Each measure requires re-synchronizing the software across the devices.

### 4.3. Hardware and Software Alternatives

The preliminary development steps and checks lead to three software–hardware *alternatives* as candidates for the final evaluation detailed in Section 4.4. The *configurations* are chosen such that they represent opposite ends of the spectrum: the Nano is a powerful computing platform, with a selection of connectors and a large heat sink, therefore being larger and heavier than the Raspberry Pi. The Pi is a fraction of the *Size* and *Weight* of the Nano, however the decreased *Size* comes at the expense of connectors and processing power. The *alternatives* are shown in Figure 2.

The baseline *alternative* is the Nano real-time *alternative*, shown in the top left of Figure 2. The nodes running on the Nano are framed in pink in the bottom row of Figure 1, while all other nodes run on the host PC. This is equivalent to a distributed system where hardware-near and higher-frequency processing is done on the onboard computer, while higher-level, lower-frequency computational tasks are offloaded [6,40].

The high computational capacity of the Nano makes the system overpowered for these reduced tasks, therefore the other two *alternatives* shown in Figure 2 are evaluated to explore diverging specifications:

1. Can the computational load of the onboard computer be increased, allowing for a higher level of autonomy, without a detrimental impact on its performance [6]?
2. Can a smaller computer be used, allowing for a lighter payload and improved flight mechanics [27]?

These three *alternatives* are evaluated with the same experiments detailed in Section 4.4, after the development steps of Figure 6 are completed.

### 4.4. Profiling

Profiling—passive recording of system performance parameters—is undertaken with an independent ROS package. The official rosprofiler package [41] is updated to fit current psutil methods and message fields are adapted. Specifically, power logging, if available on the host, is included; CPU load is consolidated across all cores; virtual memory (used and available) as well as swap (used and available) are included. The package queries the host OS through psutil at a frequency of 5 Hz to collect samples. The number of samples, the average, the standard deviation, and the maximum or minimum—whichever is more conservative—over the samples is published at a frequency of 0.5 Hz. A client node is added, which is running on the notebook and records the values matching a predefined list of hosts and nodes. It writes the values to spreadsheet file at the end of each simulation run.

#### Postprocessing

The first and last five recorded values (equivalent to ten seconds each) are omitted during post-processing to reduce the influence of start-up and shutdown processes. Following this step, the number of samples per message is checked. Due to the publishing and querying frequencies, the number of samples should be ~10. If it is less than 8, a fault is recorded and all values of the sample omitted from further processing. This fault indicator has become evident during the development steps detailed in Figure 6. The current draw of the Raspberry Pi cannot be recorded through the OS, and therefore the nominal current draw of the device is multiplied with the CPU load as an approximation.

Using extreme values is preferred over average values in computational evaluation and aerospace systems, to account for worst case scenarios. The values of interest are therefore the maximum CPU load, the maximum used memory, as well as the minimum available memory. The 75th percentile value is extracted, to introduce robustness against outliers. These factors were determined through the inspection of intermittent development steps as shown in Figure 6.

The values recorded need to be consolidated into singular values and compared pairwise to map between 0 and 9 [28,42], depending on what is considered to be a priority. For *Size* comparisons, the volume of an, approximately square, 3D printed enclosure is measured: V=l·w·h. Both devices are weighed including their cases. An UAV, including a battery and excluding an onboard computer, is weighed: $w_{UAV}=1322$ g. The relative increase in weight in percent, ${\%}_{w}=\frac{w_{onb}}{w_{UAV}+w_{onb}}$, is calculated. *Power* is commonly used as a proxy for flight time [6] and nominal values are used to calculate a baseline power usage $P_{0}$:

(2) $$P_{0}=\frac{U_{0}\cdot \left(I\cdot t\right)\cdot c}{t_{0}}$$

With values $U_{0}$ being the average voltage rating, calculated through $U_{0}=n_{cells}*U_{cell}$, with $n_{cells}=3$ and $U_{cell}=3.7V$, common for UAV LIPO batteries. *c* is a safety factor for battery drainage, for which 0.8 is used. (I·t) is a capacity rating in Amph for batteries, for which 4.0 is used. $t_{0}$ is a nominal flight time at hover, for which t0=13, equal to 20 min of flight time is used. As the decrease in flight time caused by the power consumption, without additional performance effects [6], is of interest, the new flight time is calculated using the additional electrical power consumption by the onboard computer calculated through $P_{e}=V\cdot I$, evaluating to

(3) $$t_{f}=\frac{U_{0}\cdot \left(I\cdot t\right)}{P_{0}+P_{e}}$$

The influence of additional *Weight* on the flight time is omitted, because its influence is considered marginal in comparison to the relative decrease in flight time through additional power consumption $\Delta t=\frac{t_{0}-t_{f}}{t_{0}}$, which serves as a reasonable first approximation. The power draw of the device is either calculated through multiplication of the nominal voltage with the recorded current.

Computational performance is determined through the evaluation of *Resource Utilization*, *Co-Existence*, and *Maturity*. For *Resource Utilization*, CPU load, and memory usage are recorded.

For *Co-Existence*, available memory is recorded and CPU capacity calculated through 1− CPU load. Memory values are recorded as percentages of the nominal system capacity, with the capacity of the Nano being 4096 MB and the Raspberry Pi being 512 MB. These values are used for the comparison.

If the faults recorded are either 0 or 1, their value is considered equivalent. Otherwise, the logarithms are used as inputs for relative importance.

Figure 4 depicts the final hierarchy, which is to be used for the evaluation. The first level is derived from values common across literature. The second level is based on the *SQUARE* family [31] and the third level are common values from computational architectures [36].

### 4.5. Case Studies

#### 4.5.1. Case 1—Oil Rig Inspection

**Figure 7:**
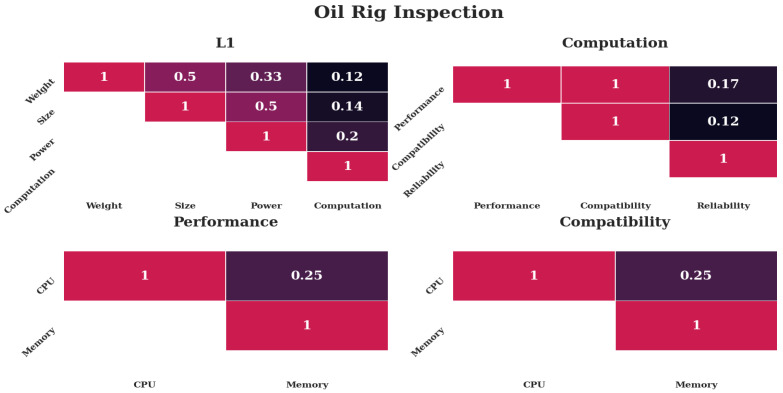
Weights put into the analysis for the case of an oil rig inspection. The top row shows the first two levels, the bottom row the third level. Only the upper diagonal weights are shown for simplicity. Darker colors indicate less preference, lighter colors more preference.

The weights for the case of oil rig inspection are shown in Figure 7. The following assumptions lead to these weights; *Computation* would be most important, with *Power* following, due to the relationship uncovered by Boroujerdian et al. [6]. *Size* should be less important, albeit more important than *Weight* due to limited space. Reliability would be most important, as failures could have catastrophic consequences. Memory should be considered more important than CPU for both cases, because this would likely be the constraining factor for system updates in the distant future.

#### 4.5.2. Case 2—Suspended Arm

**Figure 8:**
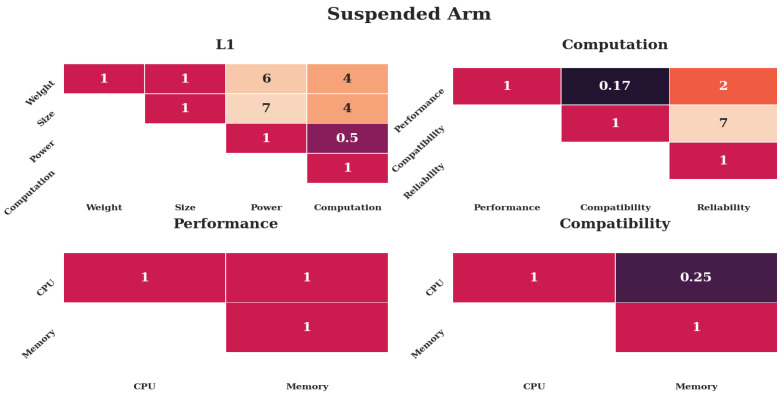
Weights put into the analysis for the case of a suspended arm. The top row shows the first two levels, the bottom row the third level. Only the upper diagonal weights are shown for simplicity. Darker colors indicate less preference, lighter colors more preference.

The weights for the assumed case of a suspended arm in a controlled environment are shown in Figure 8. *Size* and *Weight* should be considered most important, because the suspended arm should yield a large increase in *Weight*, coming close to legal limits. *Power* should be considered least important, as the controlled environment would allow for quick exchange of batteries. On the computational level, compatibility should be considered most important, because the control of the arm would require implementation space. Reliability should be considered least important, as motion capture systems could serve as a fallback. Memory should be considered more important than CPU, as the control of the arm could require additional software tools and slower CPU cycles could be compensated through time spent at hover.

#### 4.5.3. Case 3—Additional Module

**Figure 9:**
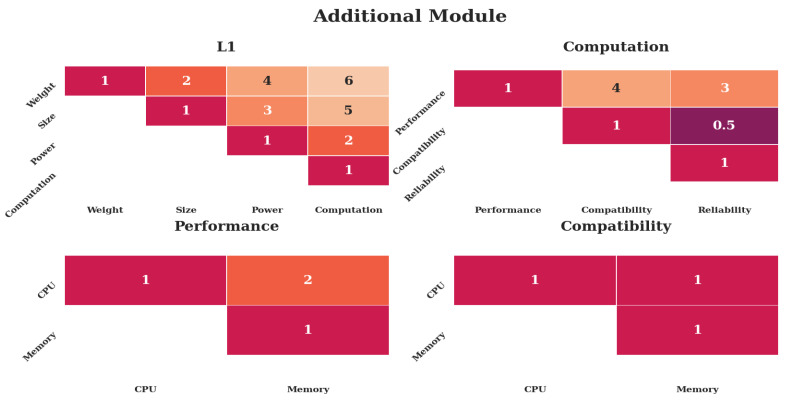
Weights put into the analysis for the case of an additional developed module. The top row shows the first two levels, the bottom row the third level. Only the upper diagonal weights are shown for simplicity. Darker colors indicate less preference, lighter colors more preference.

Figure 9 shows the weights for the case of an additional module placed on an UAV. The assumptions are made that a module would have strict requirements on *Size* and *Weight*, because it would add to the total load. *Power* would be moderately important. Performance would be more important than compatibility, since the hardware would be entirely dedicated to the software module. Therefore, requirements on free space would be secondary, as would reliability, as the module would be considered nonessential. CPU performance would be more important than memory, as CPU load would likely be the first bottleneck.

## 5. Results

Preliminary development steps, detailed in Section 4.2 and shown in Figure 6, are the reason for the following changes compared to SITL. First, the image type is changed to a compressed representation due to networking effects [21,22], which causes an increase in CPU load. Second, the MAVROS node responsible for translating between ROS and Mavlink is required to run on the onboard computer, increasing its load. Third, buffer sizes are increased for all messages except imaging. Fourth, the serial port speed is increased to 921,600 to allow for background messaging, increasing susceptibility to transmission errors. Fifth, the system load background usage through OS services is decreased. The final *configuration* for experiments are documented in Table A1 in Appendix A.

### 5.1. Profiling

Table 1 shows the values collected across the ten simulation runs exemplified by Figure 3. The values are collected for each of the three variations shown in Figure 2 and postprocessed according to Section 4.4.

The computational load is near the maximum across all devices, while the used memory is scaled according to the capacity of the device. The available memory of the Pi is much lower, in absolute as well as relative values. These effects can be explained by OS scheduling and paging techniques beyond the scope of this work. Current draw appears to be near the limits as well, which were recorded on the Nano and approximated on the Pi. The number of faults show a great variation. The Pi displays a substantial increase in number of faults compared with the Nano, while its *Size* and *Weight* are one order of magnitude lower than the Nano’s.

### 5.2. Case Studies

**Figure 10:**
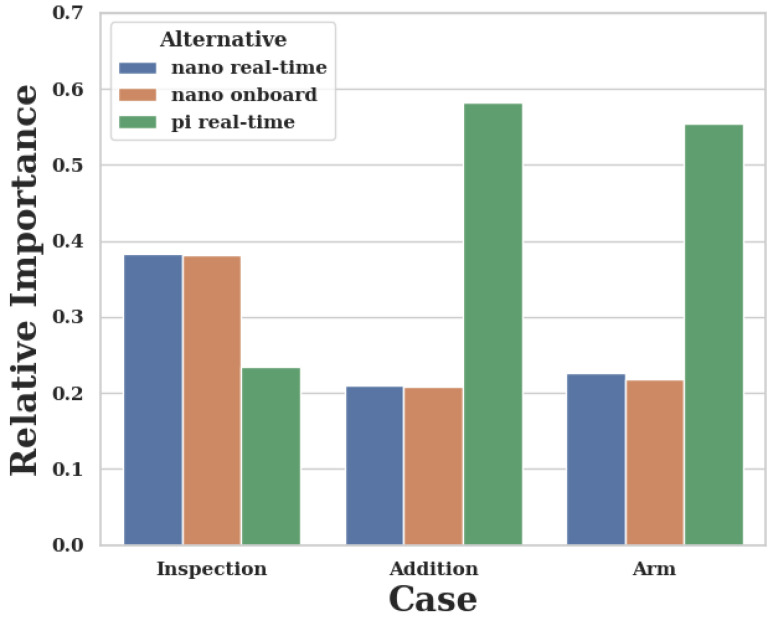
Relative preference for the case studies. The *y*-axis shows the relative preference, the *x*-axis a bar for each *alternative*: green for the Pi-real-time, blue for the Nanoreal-time and orange for the Nano-onboard *alternative*.

Figure 10 and Table 2 show the results of the case study. The first case of oil rig inspection leads to the result that the Nano in a real-time *configuration* is preferred over an onboard *configuration*. This can be explained through the omission of networking effects, which are addressed in the literature [21,22]. The second and third case show that the Pi can be a highly preferred *alternative* under relative priorities, despite the large count of faults. The Pi is a better *alternative* when mechanical requirements prevail, as described in Section 4.5.

The first case, as shown in Figure 7, places an emphasis on *Power* and *Computation*, specifically reliability. These are the characteristics where the Nano has better values. Furthermore, free memory space leads to higher preference. The almost identical values are explained with the only slight difference in CPU and memory values (see Table 1), which only impacts the third level.

The second case as shown in Figure 8 emphasizes the importance of *Size* and *Weight*. This focus on values where the Pi is on another order of magnitude than the Nano renders the increased computational performance of the Nano almost negligible.

The same can be said about the third case, shown in Figure 9, albeit a little less pronounced. The increase in importance of performance on the second level and subsequent focus on CPU performance are rendered irrelevant through the high-level focus place on *Size* and *Weight*.

### 5.3. Monte Carlo Simulation

For each of the subsystem combinations listed in Table 1 and shown in Figure 2, there will be a case where the weights result in that combination being the most suitable *alternative*. The tabulated values for each of these alternative cases can be found in Appendix B. These cases are presented as follows.

#### 5.3.1. Nano-Onboard

**Figure 11:**
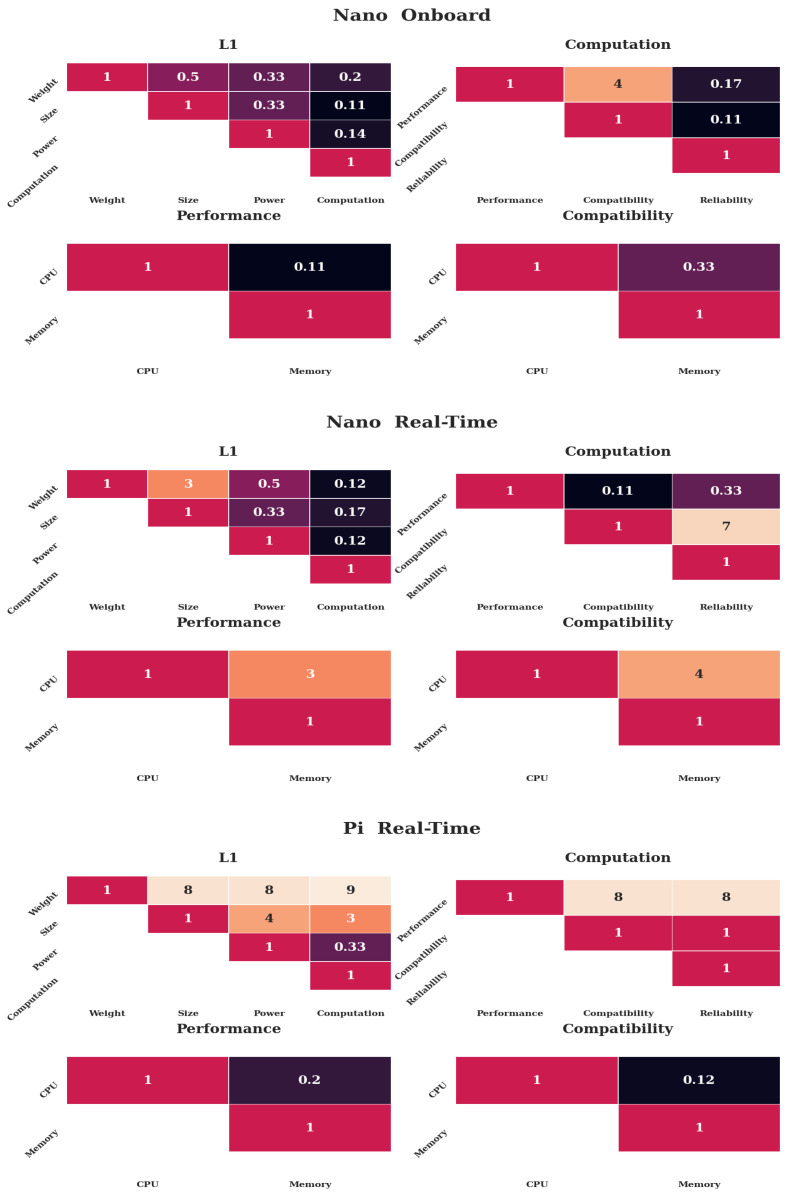
The weight matrices leading to the highest preferences for each *alternative*. The first heat maps show the weights for the Nano-onboard-*alternative*, the second set of heatmaps the weights for the Nano-real-time-*alternative*, and the third row the weights for the Pi-real-time *alternative*. The top row shows the first two levels, the bottom row the third level. Only the upper diagonal weights are shown for simplicity. Darker colors indicate less preference, lighter colors more preference.

The first four heat maps of Figure 11 show the weights required to lead to a preference of the Nano-onboard *alternative*. The preference value is 0.37926, slightly above a third. The values indicate that the Nano is preferred over the Pi, however only a slight preference was given to the full load on the Nano over the partial load. The first level shows that *Computation* receives the highest preference compared to all others. The second level indicates that reliability is considered most important, with performance being the second most important. The third level shows that memory usage is very much preferred over CPU usage and memory space a little over CPU.

These values indicate that when mechanical parameters are not as important as computational and reliability plays a significant role, the Nano dominates the decision, as expected.

#### 5.3.2. Nano-Real-Time

The preference value of the Nano in a real-time setting is 0.42533. This result indicates that the Nano in a real-time setting has quite a large preference over the other *alternatives*. The weights that lead to this preference are shown in the second set of Figure 11. The first level shows that *Computation* has the highest priority, followed by *Power*, while the other two are considered less important. The second level demonstrates the benefit of available space, which has a significantly higher value than reliability and lastly performance.

#### 5.3.3. Pi-Real-Time

The last set of heatmaps in Figure 11 shows the weights leading to the highest preference for the Raspberry Pi-real-time *alternative*. The relative preference value is 0.64268, more than three times as high as the other two *alternatives* as shown in Appendix B. Inspecting the first level reveals that *Weight* is considered most important, and *Size* second most, while *Power* and *Computation* are considered relatively less important. Computational parameters have little significance through this; however, performance largely outweighs compatibility and reliability. On the third level, memory is preferred over CPU.

## 6. Discussion

### 6.1. The AHP

The AHP is a model mainly used for high-level decision-making and has both benefits and drawbacks. On one hand it is constrained by its scale and the required consistencies [47], as well as the subjectivity of the choice of weights by stakeholders. On the other hand, the scales and consistencies force users to put their criteria into numerical values by comparing pairwise and checks whether the criteria can be considered consistent. Users are required to re-evaluate their criteria if these are deemed inconsistent and increased dimensions allow for higher degrees of inconsistencies, which are more likely to arise.

The hierarchy allows to be extended or reduced. For example, *Size* could have included an additional parameter for the form factor (for example, cubic as bad, but flat as a good form factor). Alternatively, other characteristics of the ISO, as mentioned in Section 2.1, would require consideration if *alternatives* would differ with respect to these characteristics, for example, if a solution not based on ROS would be provided, the equalities mentioned in Section 2.1 would not hold. However, the hierarchy reduces the influence of lower levels on the higher levels, which is indicated through the limited influence of reliability, despite the evident shortcoming of the Pi. Shifting values between levels may result in different outcomes, however the model was chosen in compliance with contemporary literature and the ISO model. The question whether to place reliability on a higher level should be evaluated with respect to the decomposition of structurally similar indicators as described by Saaty [28], as well as the inclusion into the ISO [31]. Weights on the third level are diluted through the higher levels and have partial co-dependence, as indicated by the inverse relation of CPU used and CPU free. Additionally, the use of ROS limits the freedom of software optimizations [36] for developers, paying the price of flexibility for reduced complexity.

The complex structure of the problem [6,27] would render common optimization techniques difficult to be applicable and is beyond the scope of this work. Unlike convex optimization or gradient-based methods, the AHP does not yield absolute performance optima; however, it provides relative preferences on readily available *alternatives* that can be compared with each other. Furthermore, the evaluation disregards hardware settings that might have an influence, such as cable shielding or memory card wear.

The research could also be extended to further *alternatives*, as only three, with two comprising of the same hardware, were evaluated. However, with an increase in *alternatives*, final preferences become less pronounced due to the mathematical normalization inherent to the AHP. Furthermore, the *alternatives* were chosen such that they represent opposite ends of the spectrum: the Pi as a mechanically lightweight *alternative*, with little computing power and the Nano as a comparably large and powerful module. The variation between the onboard and real-time *configurations* is used to illustrate differences in software load, albeit the effects are less pronounced. This can be explained through smart OS priority and assignment schemes for memory and CPU, leading to marginal differences for these aspects.

### 6.2. Case Studies

The case studies, inspired by remote-sensing scenarios, indicate that varying requirements lead to different results which *alternatives* are preferred. While the step of decreasing mechanical *Size* or increasing *Computation* may trigger a logical choice in experienced engineers, evaluating the benefits may be difficult for other users.

Networking effects are omitted, because these were addressed in the literature [21,22] and with the deployment of ROS2. However, network settings hold the potential to impact decisions, as they have an influence in UAV remote sensing applications.

The case studies are used to illustrate the influence of relative weights and how these can differ on a case-by-case basis, however the tasks presented do not necessarily correlate with the task defined in the original work [35]. Nonetheless, the methodology serves its purpose of illustrating the effect that different weights have on a final preference.

### 6.3. Monte Carlo Simulation

The MC simulation compares a large variety of different weights as inputs, combined with the same results from profiling, as shown in Table 1. This exposes the relative weights, which could have led to the preference of an *alternative*. While these results are indicative of the relative weights which could have to the preference of an *alternative*, the AHP is not used to select weights. This would contradict the paradigm of the AHP that relative weights require assignment, and not *alternatives*. The method also depends on results from profiling and therefore a variation in recording performance values may lead to a change in preference. It also poses the questions whether shifting around certain aspects to other levels, such as reliability to the topmost level, should not be considered to ensure an increased focus.

### 6.4. Comparing Case Studies and Monte Carlo Simulation

The case studies are inspired by three remote-sensing scenarios and result in different preferences, as indicated in Table 2 and Figure 10, despite being run with the same performance indicators as shown in Table 1. Figure 7, Figure 8 and Figure 9 indicate that the distinctiveness of the first level has a significant influence on the outcome. The mechanical properties *Size* and *Weight* are considered more important for the case of an additional module (Figure 9) as well as the case of a suspended arm (Figure 8), which are also the cases which give preference to the Pi, as shown in Appendix B.

The dominance of the Pi under consideration of mechanical properties is underlined by the MC results as shown in Figure 11 and Appendix B, where the first level of the Pi real-time result is predominantly focused on *Weight*. The other two alternatives are almost equal, as their mechanical properties are equal and the influence of *Computation* is diminished. When *Computation* is considered important, the Nano prevails, albeit not by as large a margin as the Pi (see Appendix B), due to the reduced benefits arising from the lower levels.

Altogether the Nano shows considerable benefits when *Computation*, and foremost reliabilitity, is important, while the Pi prevails when *Size* and *Weight* are required to be small. However, the inclusion of reliability into the computational level may lead to be misleading for fully autonomous missions and may require researchers to ensure other safety measures are in place. Generally the weights tend to shift towards mechanical importance when the configuration is not considered mission-critical and safety can be guaranteed through other measures. If basic flight depends on the configuration, computational factors gain influence, which is in agreement with contemporary research [6,27].

## 7. Conclusions

This research details a method for evaluating and selecting suitable hardware for the hardware-in-the-loop development step for deployment of remote-sensing UAVs. While computational performance is often considered secondary, it has a significant influence on which choice of software–hardware combination should be considered as suitable for a given task. A HITL software package was tested under three different software–hardware setups and profiled to collect performance indicators. The indicators are used in a decision-making model alongside subjective preference values to show how changes in priorities lead to preference of different software–hardware combinations. A Monte Carlo simulation indicates weights under which one of the *alternatives* shown in Figure 6 would be preferred.

The results provide insights into the complex decisions required to deploy autonomous algorithms on hardware and require careful selection of the preference weights as they rely on subjective assignment. However, preference weights only require ordinal-scaled definition and are checked for mathematical consistency, therefore allowing awareness for priorities of potentially conflicting *alternatives*.

The methodology presented in this research can enable remote-sensing researchers to evaluate whether their choice of software–hardware combination is sustainable by running hardware-in-the-loop simulations. This can lead to a reduced failures, improve performance consistencies, and reduce development time, improving deployment of sophisticated UAV-based remote sensing technology.

## Figures and Tables

**Table 1:** Profiling results for the three alternative cases presented in Figure 2.

Device	Nano		Pi	
Load	RT	Onb	RT	Unit
**CPU Load Max**	82.9	91.3	94.5	%
**Used Mem Max**	547	966	218	MB
**Avail Mem Min**	3249	2830	154	MB
**Current**	3.981	4.233	3.689	A
**Faults**	1	0	310	#
**Volume**	0.4464	0.4464	0.0672	l
**Weight**	0.252	0.252	0.044	kg

**Table 2:** Relative preferences for each *alternative* for the three case studies. Higher values indicate higher preference. Bold numbers indicate which alternative is preferred/the best for each column.

Device	Nano		Pi
Load	RT	Onb	RT
**Inspection**	**0.3832**	0.3818	0.2350
**Suspended Arm**	0.2267	0.2187	**0.5546**
**Add. Module**	0.2095	0.2087	**0.5828**

**Figure A1:**
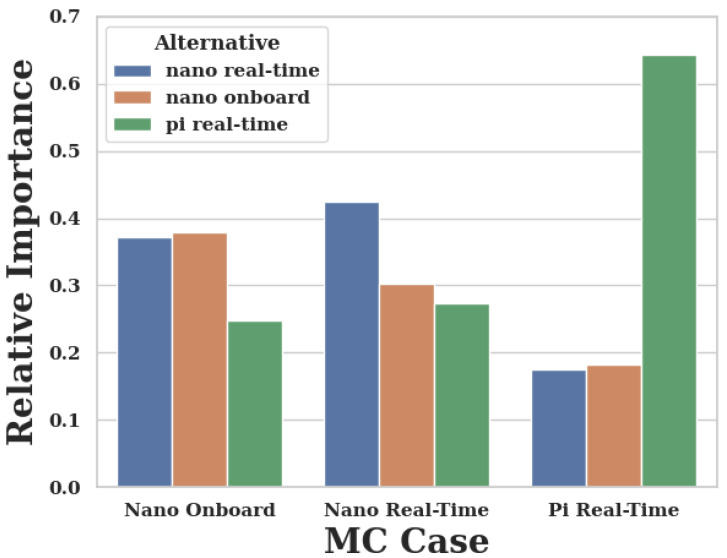
Relative preference for the Monte Carlo simulation. The *y*-axis shows the relative preference, the *x*-axis a bar for each *alternative*: green for the Pi-real-time, blue for the Nano-real-time and orange for the Nano-onboard *alternative*. Each MC results in one of the alternatives being preferred.

## Appendix A. Hardware Implementation Details

**Table A1:** Configurations of the two embedded hardware devices following initial experiments.

Device	Nano		Pi	
Software	Type	Installation	Type	Installation
**OS**	Jetpack Ubuntu 18.04	Etcher	Ubuntu Mate 18.04	Cross-compilation Pi 3B
**Swap**	Swap	2048 MB	Zram/Swap	256/1052 MB
**ROS**	Melodic	Package Manager	Melodic	Package Manager
**MavRos**	0.33.4	Source	0.33.4	Source
**OpenCV**	3.4.6.	Source + Contrib	3.4.6	Source + Contrib Cross-compilation
**Timesync**	NTP	ntp.qut	NTP	ntp.qut
**Proprietary** **Changes**	Power Mode	0	Serial Clock speed	Elevated-mavros
	HDMI	disabled	HDMI	disabled
	WiFi	Powersaving disabled	WiFi	Powersaving disabled
	Power Supply	Barrel Jack 4A	Display Service	disabled
	SD-Card	32 GB SanDisk SDHC U3	SD-Card	32 GB SanDisk SDHC U1
	Serial Port Config	921600 8N1	Serial Port Config	921600 8N1
			Bluetooth	disabled-Serial Port

## Appendix B. Monte-Carlo Simulation Results

**Table A2:** Results from the Monte-Carlo Simulation. Each row shows the global preference of the alternatives. The rows indicate the case for which these values were the highest absolute preference. Bold numbers indicate which alternative is preferred/the best for each column.

Device	Nano		Pi
Load	RT	Onb	RT
**Nano-RT**	**0.42533**	0.30175	0.27292
**Nano-Onb**	0.37235	**0.37926**	0.24840
**Pi-RT**	0.17570	0.18163	**0.64268**
